# Polymorphic edge detection (PED): two efficient methods of polymorphism detection from next-generation sequencing data

**DOI:** 10.1186/s12859-019-2955-6

**Published:** 2019-06-28

**Authors:** Akio Miyao, Jianyu Song Kiyomiya, Keiko Iida, Koji Doi, Hiroshi Yasue

**Affiliations:** 1 Institute of Crop Science, National Agriculture and Food Research Organization, 2-1-2, Kannondai, Tsukuba, Ibaraki, 305-8518 Japan; 2 Tsukuba Gene Technology Laboratories Inc, 6-320, Arakawaoki, Tsuchiura, Ibaraki, 300-0873 Japan

**Keywords:** NGS, Mutation, Polymorphism, Indel, SV

## Abstract

**Background:**

Accurate detection of polymorphisms with a next generation sequencer data is an important element of current genetic analysis. However, there is still no detection pipeline that is completely reliable.

**Result:**

We demonstrate two new detection methods of polymorphisms focusing on the Polymorphic Edge (PED). In the matching between two homologous sequences, the first mismatched base to appear is the SNP, or the edge of the structural variation. The first method is based on *k*-mers from short reads and detects polymorphic edges with *k*-mers for which there is no match between target and control, making it possible to detect SNPs by direct comparison of short-reads in two datasets (target and control) without a reference genome sequence. The second method is based on bidirectional alignment to detect polymorphic edges, not only SNPs but also insertions, deletions, inversions and translocations. Using these two methods, we succeed in making a high-quality comparison map between rice cultivars showing good match to the theoretical value of introgression, and in detecting specific large deletions across cultivars.

**Conclusions:**

Using Polymorphic Edge Detection (PED), the *k*-mer method is able to detect SNPs by direct comparison of short-reads in two datasets without genomic alignment step, and the bidirectional alignment method is able to detect SNPs and structural variations from even single-end short-reads. The PED is an efficient tool to obtain accurate data for both SNPs and structural variations.

**Availability:**

The PED software is available at: https://github.com/akiomiyao/ped.

**Electronic supplementary material:**

The online version of this article (10.1186/s12859-019-2955-6) contains supplementary material, which is available to authorized users.

## Background

The detection of polymorphisms using short-reads generated by next generation sequencers is used in many fields, such as gene mapping, gene isolation, disease diagnosis, mutation appraisal and genome evolution. Accurate detection of polymorphisms is a prerequisite for these purposes. A common method of polymorphism detection is to align polymorphisms with reference genomic sequences using high-speed aligner programs, such as bwa or bowtie [1, 2], and then to extract polymorphic portions with filter programs such as Samtools and GATK [3, 4]. These processes have been used in most studies for next generation sequence analysis, and, therefore, are considered to be the de facto standard [5, 6]. Because they are designed to detect the maximum number of polymorphisms, the results contain a non-negligible number of false-positives. To eliminate false-positives, combination with other techniques, such as microarrays is adopted in these NGS analyses.

Detecting polymorphisms without using these aligner programs is interesting for verification of results from the de facto standard methods. When comparison is made between two sequences from homologous parts and a first mismatched base is detected, the mismatched base is considered to be the ‘polymorphic edge’ between the two sequences. We have devised two methods to detect these polymorphic edges. One method is to detect the polymorphic edge directly from both target and control sequence by dividing them into *k*-mers and comparing them, which detects primarily SNPs. The other method is to detect the mismatched bases by ‘bidirectional alignment’, i.e.*,* comparing the target reads against a reference genome sequence from both ends of the target reads. This process makes it possible to detect not only SNPs but also insertions, deletions, inversions, and translocations. The polymorphisms detected by both methods are verified by counting target and control reads containing the polymorphisms. The verification process developed in this study provides an accurate detection of polymorphisms. Using our methods, we obtained high quality polymorphisms from NGS sequence of rice. Comparison maps of SNPs between two rice cultivars clearly shows chromosomal segments which were introduced during breeding without additional selection or filtering of polymorphisms. In addition, the ‘bidirectional method’ detects specific large-deletions among cultivars. The PED is an efficient and accurate tool for genotyping polymorphisms using next generation sequencing data.

## Results

### *K*-mer method to detect polymorphic edges

**Fig. 1:**
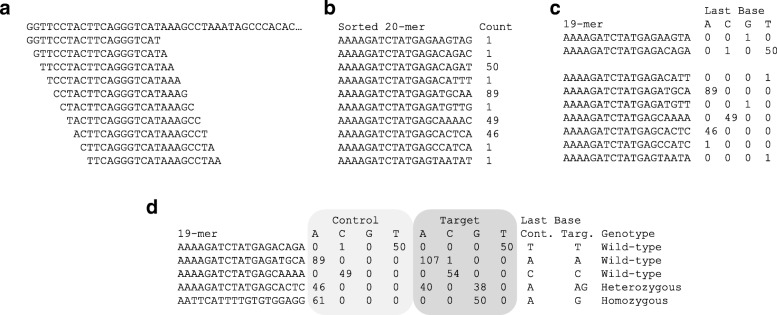
Polymorphic edge detection by *k*-mer method **a ***k*-mers (*k* = 20) from all positions of short reads are obtained. **b ***k*-mers are sorted and counted. **c** Sorted *k*-mers are divided into first (*k*-1)-mer and last base, and converted to (*k*-1)-mer following counts of last A, C, G and T. **d** The last-base-count data from target and control are joined by (*k*-1)-mer sequence. If the pattern of counts is different, the last base may be the polymorphic edge

The *k*-mer method is based on comparison of the bases at the 3′-ends of *k*-mers. From short-reads of target and control, *k*-mers (*k* = 20) from all positions are obtained (Fig. 1a) and all *k*-mers are sorted and counted (Fig. 1b). Because the step of counting is essentially the same as the MapReduce in Hadoop, the *k*-mer method is suitable for parallel computing for faster processing and scaleup [7]. All *k*-mers are divided to the first 19-mer and the last base. The count of the 20-mer is regarded as the count of the last base following the first 19-mer. Thus, the count data can be transformed to the 19-mer sequence and the last base counts for respective last base A, C, G, and T (Fig. 1c). The count of the respective base corresponds to the genome read-depth of short-reads containing the base. If there is no polymorphism at the last base following the 19-mer sequence, the base showing the highest count in the target should be the same as that in the control. When polymorphism exists in the last base of the *k*-mers, the last base showing the highest count in the target should be different from that in the control (Fig. 1d). Thus, this method can detect the polymorphic edge. This method can detect SNPs, but only indicates the existence of an insertion/deletion, reversion and translocation.

### Algorithm of *k-*mer method

1. *K-*mers from all positions of sequence are obtained.
2. *K-*mers are sorted and all kinds of *k-*mers are counted.
3. Counted *k-*mers are divided first (*k-*1)-mer and last base.
4. Count data are transformed to (*k-*1)-mer and counts for respective last base A, C, G and T.
5. Each transformed data set is obtained from target and control sequence.
6. Data from the target and control are joined by (*k-*1)-mer sequence.
7. If highest count base between target and control in same (*k-*1)-mer data is different or another base with high level count is found, the last base following (*k-*1)-mer is the polymorphic edge.

### Bidirectional alignment method

**Fig. 2:**
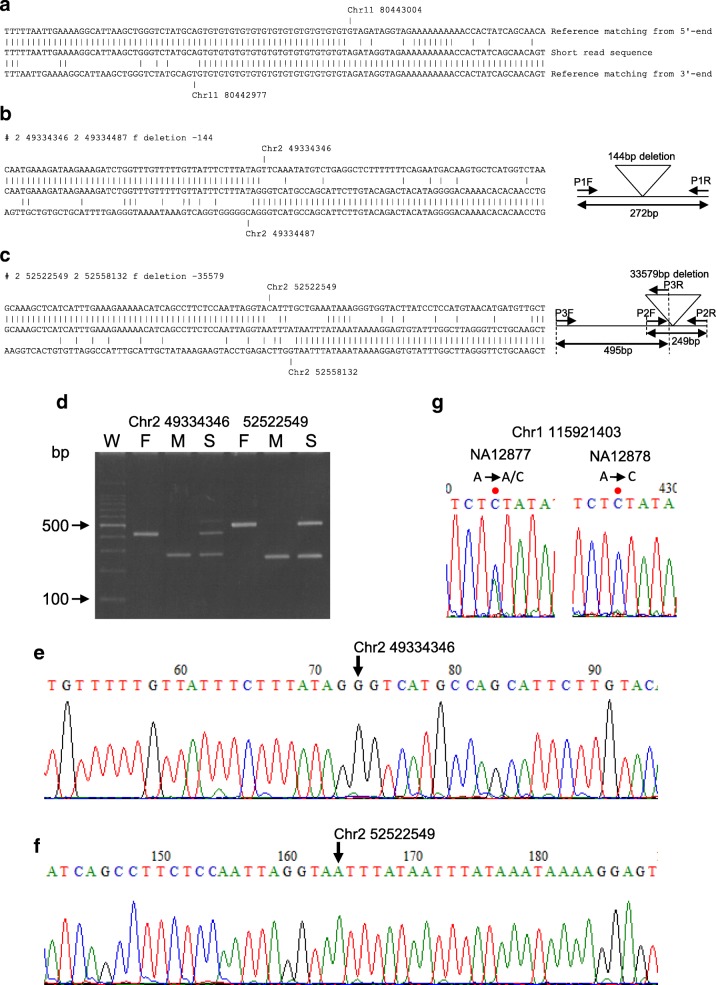
Polymorphic edge detection in human genome by bidirectional alignment **a** An example of bidirectional alignment (2 bp deletion on chromosome 11 in hg38). **b** Alignment of 144 bp deletion on chromosome 2 in hg38 and the diagram of PCR primers and amplified products for confirmation. **c** Detection of 35.6 kb deletion and the diagram for confirmation. **d** Confirmation of 144 bp (position 49,334,346 on chromosome 2) and 35.6 kb (position 52,522,549 on chromosome 2) deletion. W: molecular weight marker (100 bp ladder), F: Father (NA12877), M: Mother (NA12878), S: Son (NA12882). For detection the 35.6 kb deletion, primer pairs which amplify wild-type of father and deletion of mother are used for PCR, and the reaction mixtures are electrophoresed. **e** Detection of a junction for the 144 bp deletion. **f** Detection of a junction for the 35.6 kb deletion. **g** Confirmation of SNP at position 115,921,403 on chromosome 1, A to A/C in father and A to C in mother by Dye-terminator sequence method

Detection of polymorphic edges in human genomes by the bidirectional alignment is shown in Fig. 2a. First, both end sequences of short-reads are aligned to the genome sequence, then the alignment is subjected to analysis in the inward direction of the short-read; this enables the detection of polymorphic edges from the 5′-end and 3′-end. When the distance between the reference genome-mapped positions of the 5′-end and 3′-end of a short-read is different from the corresponding length on the short-read, an insertion or deletion is shown to exist in the short-read. Reverse direction in the alignment of a short-read end demonstrates that the short-read contains an inversion. When either end of a short-read is mapped to the genome at different chromosomes, a translocation site is shown to exist in the short read (Additional file 1: Figure S1).

### Algorithm of bidirectional alignment method

1. Search homologous region of 5′- and 3′-ends of short-read against reference sequence.
2. If distance of mapped positions is different from distance 5′- and 3′-ends, the short read has SV.
3. Align from 5′-end of the read with reference sequence. If mismatched base appears, the base is the polymorphic edge.
4. Detect the polymorphic edge from 3′-end of read.

### Detection of new SNP and deletions from platinum human genome sequence

The bidirectional alignment method can also detect large structural variations. Illumina provides human genome sequence data from the 17-member CEPH pedigree 1463 and high-confidence variant calls. We downloaded the 17-member fastq data and analyzed the fastq data by the bidirectional methods. The variants revealed by our methods were compared with the variants reported by Illumina, hg38.hybrid.vcf called by use of bwa-mem, Issac, GATK3, Free-Bayes, Platypus, Strelka, Cortex and CGTools 2.0 [8]. For example, two deletions (144 bp and 35.6 kb deletions), shown in Fig. 2b and c, were detected in mother (NA12878) by the bidirectional method, but were not detected in father (NA12877). These large deletions are absent in the hg38.hybrid.vcf from NA12878.They are for the first time described in the present study using the bidirectional method. As shown in Fig. 2d, the existence of these deletions and their inheritance to the son (NA12882) was confirmed by PCR and agarose gel electrophoresis. Detected polymorphic edges were also confirmed by Dye-terminator sequence method (Fig. 2e and f). Likewise, SNPs were detected for the first time in the present study, an example is shown in Fig. 2g and was confirmed by PCR and sequencing. In total, we detected 25,489 SNPs and 184,629 structural variations which are absent in the hg38.hybrid.vcf by the bidirectional method (data not shown).

### Comparison of performance using an artificial genome with introduced mutations at known positions

**Fig. 3:**
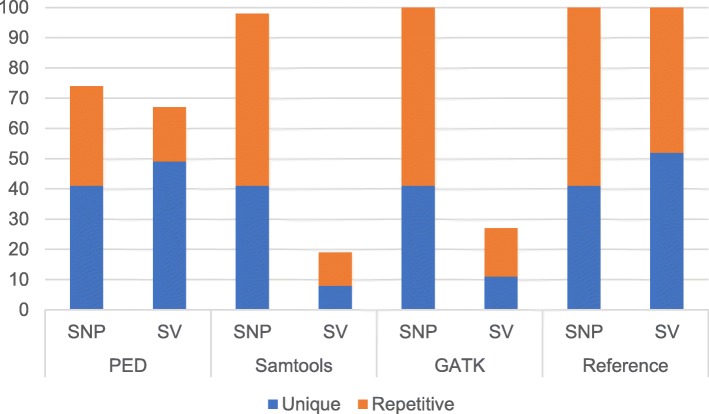
Performance of polymorphism detection. Percentage of detected polymorphisms in rice genome introducing artificial mutations. Blue; Polymorphisms detected in unique regions, Orange; detected in repetitive regions. Ratio of unique and repetitive regions of mutations were shown in ‘Reference’. SNPs detected the *k-*mer method are included in the output of bidirectional alignment method

To examine the performance of our two methods, we compared the results of our methods with those of Samtools and GATK using a reference rice genome sequence and its artificially modified sequence, which contained 54 single base exchanges and 108 structural changes (insertion, deletion, inversion, or translocation) at various positions on the chromosomes (see Methods and Additional file 2: Table S1 and Additional file 3: Table S2). Figure 3 shows the percentages of detected polymorphisms by PED, Samtools and GATK. The ratio of unique and repetitive regions at the mutations are 41 and 59% for SNPs, and 52 and 48% for structural variations. The definition of unique region is a single hit of BLAST search against the reference genome. The query from reference genome sequence for BLAST search is a 200 bp sequence containing the mutation at the center. When the result of BLAST shows multiple hits, the mutation is classified as repetitive. Because the *k*-mer method does not return structural variations and detected SNPs are included in result of bidirectional method, results from *k*-mer method and bidirectional method are combined as the result of PED. For SNP detection, all methods detect all known SNPs on unique regions, indicating all methods have no false-negatives for the unique region. On repetitive regions, 33, 57 and 59% of total SNPs were detected by PED, Samtools and GATK, respectively. For structural variations on unique regions, 49, 8, and 11%, respectively, were detected. Clearly, PED detects more structural variations than Samtools and GATK. On the other hand, for SNP detection, Samtools and GATK detect more SNPs than PED, especially in repetitive regions. The detection of polymorphism on repetitive region, especially in the case of high similarity among repetitive sequences, returns two or more positions for single polymorphism. Among them, one may be correct, and some or all of the others may be false-positives, which will interfere further analysis.

### Coverage of SNPs between bidirectional alignment method and pipeline of bwa, Samtools and GATK for graphical comparison

**Fig. 4:**
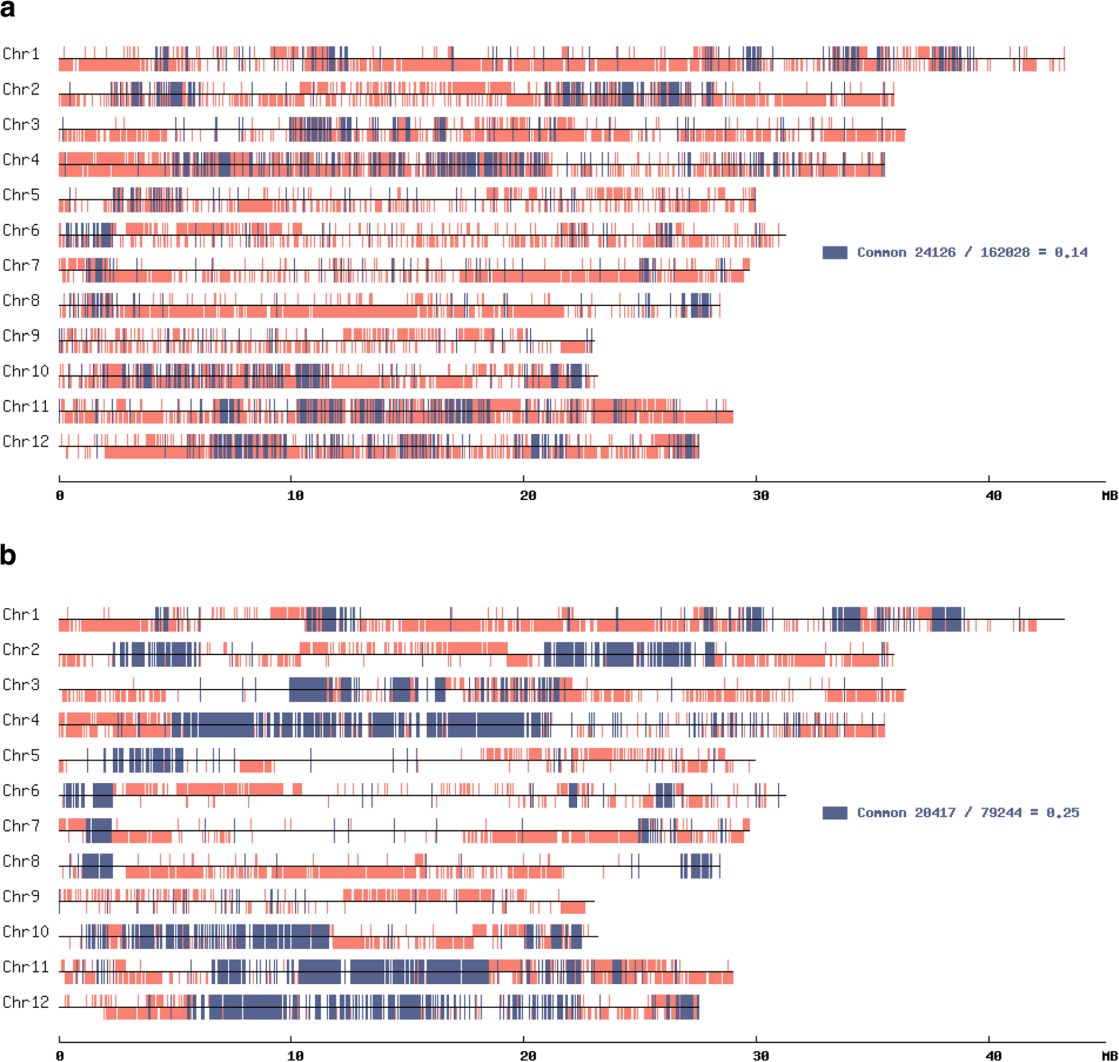
Comparison of graphical genotype of SNPs detected bwa + GATK and bidirectional alignment. **a** map of bwa + GATK data, **b** map of bidirectional alignment data. For GATK results, SNP positions which show 40 or more read depth and 1000 or more quality were selected. For SNPs detected by bidirectional alignment, SNPs marked ‘M’, i.e.*,* ‘homozygous’ were selected. Upper side of the chromosomal map is plotted with SNPs from ‘Norin8’. Lower side is ‘Koshihikari’. Common SNPs between ‘Norin8’ and ‘Koshihikari’ are indicated with dark blue. ‘Norin8’ or ‘Koshihikari’ specific SNPs are indicated with salmon pink. Regions without color indicate the same region with ‘Nipponbare’ reference genome

‘Norin8’ is the two generations backwards ancestral strain of ‘Koshihikari’. Because genome sequence of reference is from ‘Nipponbare’ which is the three generations forwards progeny of ‘Norin8’, SNPs of ‘Norin8’ and ‘Koshihikari’ against ‘Nipponbare’ genome were obtained. Homozygous SNPs of ‘Norin8’ and ‘Koshihikari’ were plotted on the genetic map. Detected SNPs of ‘Norin8’ and ‘Koshihikari’ were plotted on upper and lower sides of chromosomal map, respectively (Fig. 4). If more than 30% of existing SNPs between ‘Norin8’ and ‘Koshihikari’ within one pixel corresponding to 50,562 bp length are same, the region to the pixel has the common segment of ‘Norin8’ and ‘Koshihikari’ and is marked dark blue. If same SNPs in the pixel is less than 30%, the segment corresponding to the pixel has ‘Norin8’ of ‘Koshihikari’ specific SNPs and marked with salmon pink. Regions without the mark indicates that no SNPs for the reference genome of ‘Nipponbare’ exist in the region. Figure 4b is the plot of SNPs detected by the bidirectional method. The plot of bidirectional methods is clearer than the plot by pipeline of bwa, Samtools, and GATK (Fig. 4a). Introduced segments from ‘Norin8’ to the ‘Koshihikari’ with dark blue are easily recognized by the bidirectional method. The ratio of common SNP in total number of SNP detected by the bidirectional method, i.e.*,* ratio of inherited from ‘Norin8’ to ‘Koshihikari’ is 0.25. The value is completely in agreement with the expected value 1/4. However, in the results of the GATK pipeline, the ratio is 0.14, indicating that common SNPs may have been diluted with false-positives. Numbers of common SNPs between the bidirectional method and the GATK pipeline are almost same, indicating that the both methods detect almost the same SNPs in unique regions and false-negatives on unique regions may appear in only small amounts. Indeed, all of SNPs on unique regions of the artificial genome sequence were detected by both methods. In addition, because border of segments from ‘Norin8’, and several segments in each chromosome, are shown more clearly by the bidirectional method than by the GATK pipeline, the blurriness of map of the GATK pipeline seems to be caused by false-positives from repetitive sequences.

### Benchmark

The key technology in this study is ‘join’. Target and control sequences are sorted by sequence, and then data containing the same sequence are selected by the ‘join’ command of Unix. For example, the time complexity for a linear search of ‘m’ short-reads of the target to ‘n’ sequences of the reference is in Landau notation *O*(m n). For a binary search of short reads against the reference, the time complexity is *O*(m log n). For the ‘join’ method, the time complexity is *O*(m), because the join compares two files from head to tail, indicating that the ‘join’ method implementation could require less calculation time than other search methods. Because the ‘join’ requires pre-sorted data, sorting of the data is a bottleneck of our method. The main requirement of CPU usage and memory depends on requirement of the ‘sort’ command. Sorting is also required by bwa, Samtools and GATK. The benchmark of SNP detection is shown in Table 1. All values of runtime are SNP detection in *Caenorhabditis elegans* (ERR3063487) and ‘Koshihikari’ (DRR054198) short reads (Additional file 5: document). The runtime of the bidirectional method is at least 2.6 times faster than that of the analysis pipeline of bwa, Samtools and GATK, although the *k*-mer method is about 1.7 times slower because of the huge number of *k*-mers, i.e.*, k* × (length of read – *k*) × number of reads counted. The requirements for our two methods is much less than the requirements for the pipeline of bwa, Samtools and GATK.

**Table 1:** Benchmark of SNP detection

Method	ERR3063487	DRR054198
bwa + GATK	11,095 ± 60	154,105 ± 2761
*k*-mer	19,263 ± 22	187,177 ± 194
bidirectional	4149 ± 2	36,657 ± 309

Short reads of *Caenorhabditis elegans* (ERR3063487, 100base, 13,549,514 pairs) and *Oryza sativa* L. *cv.* Koshihikari (DRR054198, 101base, 191,991,610 pairs) were analyzed by single computer with Intel Pentium G3250 @ 3.20GHz (2 cores) and 32Gb memory. Three times run averages with standard deviations of CPU user time (seconds) by time command were shown

### Detection of ‘Koshihikari’ specific deletions among rice cultivars

**Fig. 5:**
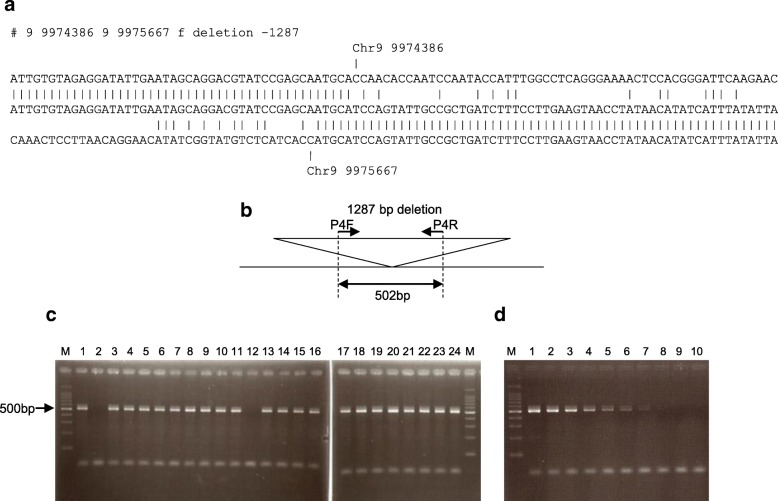
Detection cultivar specific deletion. **a** The bidirectional alignment of 1287 bp deletion in ‘Koshihikari’. The deletion does not exist in ‘Nipponbare’, ‘Aichinokaori’, ‘Akitakomachi’, ‘Hinohikari’, ‘Hitomebore’ and ‘Kinuhikari’. **b** A diagram of primers and estimated product size. **c** Detection of deleted region using primer from the deleted region. Lane M; 100 bp ladder, 1; ‘Nipponbare’, 2; ‘Koshihikari’, 3; ‘Akitakomachi’, 4; ‘IR64’, 5; ‘Aichinokaori’, 6; ‘Asahinoyume’, 7; ‘Oborozuki’, 8; ‘Kinuhikari’, 9; ‘Kirara397’, 10; ‘Koshiibuki’, 11; ‘Sainokagayaki’, 12; ‘Sasanishiki’, 13; ‘Tsugaruroman’, 14; ‘Nanatsuboshi’, 15; ‘Haenuki’, 16; ‘Hatsushimo’, 17; ‘Hanaechizen’, 18; ‘Hitomebore’, 19; ‘Hinohikari’, 20; ‘Fusakogane’, 21; ‘Massigura’, 22; ‘Yamadanishiki’, 23; ‘Yumetsukushi’, 24; ‘Yumepirika’. **d** Detection of contamination. Sequential diluted ‘Nipponbare’ DNA was mixed to 10 ng ‘Koshihikari’ template DNA. Lane M; 100 bp ladder, 1; 10 ng, 2; 5 ng, 3; 2.5 ng, 4; 1.25 ng, 5; 0.63 ng, 6; 0.31 ng, 7; 0.16 ng, 8; 0,08 ng, 9; 0.04 ng, 10; 0.02 ng ‘Nipponbare’ DNA in 10 ng ‘Koshihikari’ DNA

Our bidirectional alignment method can detect large deletions. We analyzed NGS of popular cultivars of *japonica* rice. ‘Koshihikari’ is one of the most preferred cultivars among Japanese rice farmers. To make the contamination detection system for ‘Koshihikari’, we searched ‘Koshihikari’ specific deletions from NGS data of ‘Aichinokaori’, ‘Akitakomachi’, ‘Hinohikari’, ‘Hitomebore’ and ‘Kinuhikairi’ by bidirectional alignment method [9]. The alignment on Fig. 5a is the 1287 bp large deletion which was detected in only ‘Koshihikari’ short reads. A primer pair was selected within the deleted sequence in reference ‘Nipponbare’ genome (Fig. 5b). Using the primer pair, the deleted region is examined by PCR using template DNA of 24 cultivars (Fig. 5c). Because ‘Koshihikari’ does not have the region, product was not amplified. Other cultivars except ‘Sasanishiki’ have the region and PCR products were amplified. If the DNA is from seeds of ‘pure Koshihikari’, the product will not be amplified by PCR. If seeds of other cultivars except ‘Sasanishiki’ are present as contaminants, the product will be amplified. Figure 5d indicates the amplification of template DNA of ‘Koshihikari’ with sequentially diluted ‘Nipponbare’ DNA. Contamination of even 0.15 ng ‘Nipponbare’ DNA was detected. This deletion could be used as germplasm quality control tool to detect cultivar contamination from ‘Koshihikari’.

### Detection of cancer specific SNPs by *k*-mer method

Because our *k*-mer method can detect SNPs by direct comparison of the reads between two samples, we applied direct detection of SNPs between follicular lymphoma cells (SRR2096535) and normal blood cells from the same patient (SRR2096532) [10]. No homozygous SNP was detected, while 1042 heterozygous SNPs were detected. Of these SNPs, 514 were detected in the forward and complementary *k*-mers, indicating with high accuracy that all the novel 514 SNPs were “genuine” SNPs occurring in lymphoma cells (Additional file 4: Table S3).

## Discussion

Utilizing the polymorphic edges of polymorphic regions, we developed two methods, based on *k*-mers and bidirectional alignments, to detect polymorphisms from short-reads of next-generation sequencer data. There are a lot of applications using *k-*mer. However, detection of the ‘Polymorphic Edge’ using *k-*mer is reported for the first time in this study. From the ‘direct comparison’ by *k*-mers between the two samples, we succeeded in detecting 514 novel heterozygous SNPs occurring in cancer tissue compared to normal tissue of same person. In the Additional file 4: Table S3, reads containing reference and alternative type base are detected in cancer tissue, although reads containing only reference type base are detected in normal tissue, indicating high accuracy of the *k*-mer method. The direct comparison enables detection of polymorphisms even from non-reference genome samples. In addition, *k*-mers that have polymorphic edges themselves are identifiers of polymorphism. These identifiers can be used for analysis of genetic linkage.

On the other hand, the bidirectional alignment is fundamentally different from the existing alignment, Needleman-Wunsch algorithm for global alignment and Smith-Waterman algorithm for local alignment [11, 12]. The bidirectional alignment compares nucleotide sequences from both ends of short-read sequences toward the inward sequence between target and reference genome, and finds the first mismatched positions in both directions, i.e.*,* the ‘polymorphic edges’. The pair of edges, i.e.*,* the edges detected from both directions, is able to detect not only SNPs but also insertions, deletions, inversions and translocations. The bidirectional method is similar to the ‘Split-read’ method [13]. The split-read method detects partially mapped fragments and deduces the SV from similarity of fragments. The bidirectional method returns positions of ‘polymorphic edge’ by matching from 5′- and 3′-ends of read. The pair of edges detected by bidirectional alignment enables clear detection of structural variations (Additional file 1: Figure S1).

The bidirectional alignment is fast. The key point of our methods for the performance is the ‘join of presorted sequence’. Our algorithms accelerate the analysis by making a fast ‘join’ between presorted data. Although the process is not a comprehensive string ‘search’ which would require more memory and CPU resources than our method, the PED process returns the same result.

The scope of detection is the length of the *k*-mer for the *k*-mer method and the read length for the bidirectional alignment method. On the other hand, the scope of bwa working with Samtools and GATK is the length of short read. The bidirectional alignment requires the position on the genome for both ends of the short-read. When the 5′- or 3′-end of a short-read has a repetitive sequence, the determination of positions at both ends on the genome and subsequent bidirectional alignment is difficult. However, because bwa often returns mapped positions even on the repetitive regions, the pipeline of bwa, Samtools and GATK outputs locations of polymorphisms even in repetitive regions. The total amount of detected polymorphisms by our methods is less than the pipeline of bwa, Samtools and GATK due to poorer detection on repetitive regions. Mapped polymorphisms on repetitive regions often interfere with the detection of correct polymorphisms.

The simulated SNP experiment with artificial short-reads from a rice genome containing artificial mutations at fixed positions revealed that the bidirectional alignment method had a higher detection performance for large deletions, inversions and translocations than Samtools or GATK.

When comparing genotypes graphically, we chose the threshold for GATK data selection to obtain about the same number of ‘common’ SNPs as in the bidirectional method. The high-resolution genetic map by the bidirectional method is much clearer, with inbreeding coefficient is 0.25, than by pipeline of bwa, Samtools and GATK, with inbreeding coefficient is 0.14, indicating that SNPs detected by the pipeline includes a considerable amount of false positives as a result of overly sensitive detection on the repetitive regions.

In this study we developed the germplasm contamination detection system using ‘Koshihikari’-specific large deletion selected by the bidirectional method. Because the primer pair for amplification is designed within the deletion of reference sequence, the PCR product will not be amplified with ‘pure Koshihikari’ DNA as a template. The system is highly sensitive, as it can detect the seed contamination from other cultivars with only 0.15 ng contamination in the DNA sample of 10 ng Without our bidirectional alignment method, detection of the specific large deletions will be difficult using other methods. Previously, identification of a cultivar was made by a combination of multiple markers, but the detection of a small amount of contamination was impossible.

## Conclusion

In summary, the bidirectional alignment is recommended for routine detection of mutations, because the bidirectional alignment method is faster than the *k*-mer method and can detect insertions, deletions, inversions and translocations which cannot be detected by the *k*-mer method. On the other hand, the *k*-mer method does not require a reference genome sequence at the stage of polymorphism detection. The pair of (*k*-1)-mer sequence and the subsequent polymorphic base itself is used as the identifier of polymorphism and genotype. These identifiers can then be used as markers for association analysis between genomic/cDNA sequences and phenotypes.

## Methods

### Reference genome sequence and short-read sequence

The rice genome sequence, IRGSP1.0, was obtained from https://rapdb.dna.affrc.go.jp/. The human genome sequence, hg38, was obtained from http://hgdownload.soe.ucsc.edu/goldenPath/hg38/bigZips/hg38.fa.gz.

Short-read data with fastq format were downloaded from the NCBI Short-Read Archive by ‘fastq-dump --split-files -A Accession’. Fastq data of Illumina platinum genomes from the 17 members CEPH pedigree 1463, NA12877 (ERR194146), NA12878 (ERR194147) were used. For detection of SNPs in cancer tissue, SRR2096532 and SRR2096535, public data of Texas Cancer Research Biobank Open Access Data Sharing BioProject, were used. For SNP and deletion detection of rice, Koshihikari (DRR054198), Norin8 (DRR003659), Hitomebore (DRR099983), Sasanishiki (SRR1016470 and SRR1016471) were used. For comparison of runtime, fastq data of *Caenorhabditis elegans* (ERR3063487) were used. High-confidence variant calls for NA12878, hg38.hybrid.vcf, were obtained from the Illumina website of platinum genomes (https://www.illumina.com/platinumgenomes.html). The following DNA samples were obtained from the NIGMS Human Genetic Cell Repository at the Coriell Institute of Medical Research: [NA12877, NA12878, NA12882].

### Excluding PCR bias in short-reads

Short-reads which do not contain ‘N’ were selected. Sequences of selected reads and their complimentary sequence were sorted, and then duplicated reads were removed by the uniq command of Unix. The data on sorted unique reads were used for further analysis.

### Sorted *k*-mer from sorted unique reads

From the sorted unique reads, *k*-mers (*k* = 20) at all positions of the reads were sliced (Fig. 1a). The *k*-mers were sorted by sort command and then all kinds of *k*-mers were counted by the ‘uniq -c’ command (Fig. 1b).

### Conversion to the last-base count

The *k*-mer count was recognized as the frequency of the last base of the *k*-mer described by the first 19-mer, *i. e.,* (*k*-1)-mar. All pairs of *k*-mer and count were transformed to the first 19-mer and counts of A, C, G, and T of the last base (Fig. 1c).

### Direct detection of SNP comparing (*k*-1)-mer and counts of last base

Two files of the last-base count from short-reads of the control and target were joined by the same 19-mer using Unix command ‘join control target’. The control and target counts were followed by the 19-mer sequence (Fig. 1d). If the last base was polymorphic, different bases with significant high counts between control and target will be detected. The polymorphic base could be identified by the sequence of the first 19-mer.

### Sorted *k*-mer of reference sequence for mapping

All positions of the *k*-mers (*k* = 20) and their complimentary sequences from the reference sequence were output with an order of *k*-mer sequence, chromosome number, position and direction. All data were sorted by *k*-mer sequence, and then data of unique *k*-mer were selected. The name of data file is ‘reference’.

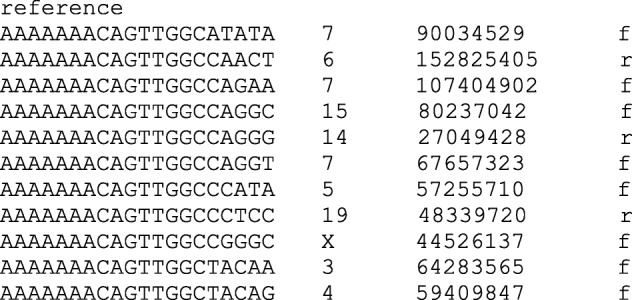



### Mapping by join command

Because the ends of short-reads tend to have relatively low quality, *k*-mers at 5 bp inside of the short-read were selected, and output with the order of the *k*-mer sequence, entire short-read sequence and start position of the *k*-mer. Data were sorted by *k*-mer sequence. The name of the data file is ‘target’.

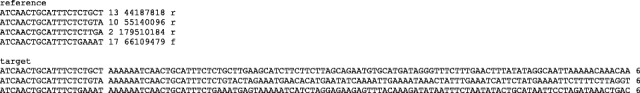



The target file and the reference file are joined by the ‘join’ command of Unix.

‘join target reference’.

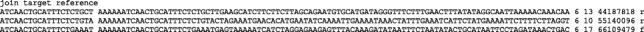



Joined data had a mapped position at 5 bp inside of the short-read. The positions of *k*-mers at the 3′-end of the short-read were obtained in the same way with the same size of margin. Following is an example of a mapping result with 10 bp margins.

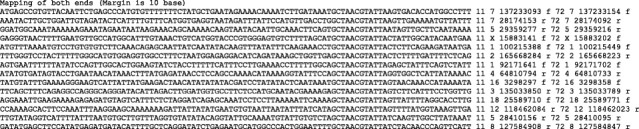



### Detection of polymorphic edges by bidirectional alignment

Mapped short-reads (middle strand) were aligned with reference sequences from both the 5′-end (top strand) and 3′-end (bottom strand), and mismatches as polymorphic edge were detected (Fig. 2a). To determine the ‘edge’, when the first gap appeared during alignment, the following five bases were aligned. If the following five bases contained two or more mismatches, the first position of the gap was assigned as the ‘edge’.

### Verification of mutations

Detected SNPs were listed with the order of chromosome number, position, reference base, alternative base, and number of supported reads.

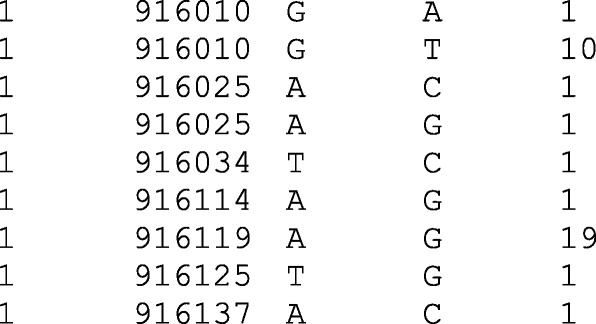



All combinations of sequences containing the SNP position (underlined, shown below) were sliced from the reference sequence. The sliced length was same as the target short-reads. The sequence changed to the alternative base (underlined) was also output. The data format was the order of sequence, chromosome number, position, reference base, alternative base, tw (for target, wild-type) or tm (for target, mutation). All data were sorted by sequence. The sorted files were used as the validation files for mutations.

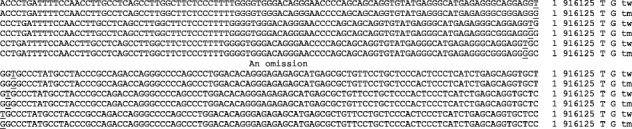



From the validation file, data contained in target reads were selected by the Unix ‘join’ command.

join target.sort_uniq validation_file.

Following is the selection result.

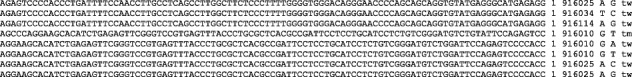



The first column of the sequence was removed, and then the appearance of data in the remaining columns was counted using the following command.

awk ‘{print $2, $3, $4, $5, $6}’ selected_result |sort | uniq -c.

An example of output is the following.

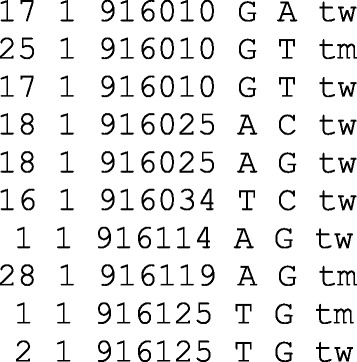



At position 916,010 on chromosome 1, 17 short-reads containing wild-type ‘G’ and 25 reads containing alternative ‘T’ were detected.

Finally, the format of the verified data was the order of chromosome number, position, reference base, alternative base, number of detected reads by the bidirectional alignment, number of detected reads containing reference-type in control reads, number of detected reads containing the alternative in control reads, number of detected reads containing reference-type in target reads, number of detected reads containing the alternative in target reads, and genotype (H; heterozygous, M; Homozygous). Control reads were from the control sample or made from the reference sequence (sliced with 100 bp length at each odd position and their complementary sequence). The data with 1 or less alternatives in control reads and 1 or less reference-type in target reads were marked ‘M’ as homozygous mutation. The data with 1 or less alternatives in control reads, and in which each reference-type and alternative was between 30 and 70% in target reads, were marked ‘H’ as heterozygous mutation.

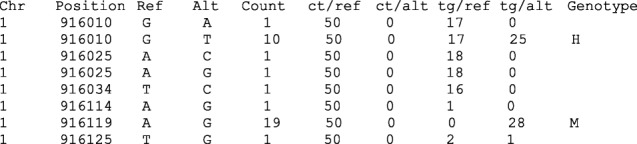



### Artificial rice genome with mutations

SNPs were introduced into each chromosome sequence of the rice genome (IRGSP1.0) with 10 Mbp intervals. In addition, 2 bp mismatches at position 10,010,000 and 10,010,001 in each chromosome were introduced. For structural variation, 1 bp, 10 bp, 100 bp deletions, 1 bp, 10 bp, 20 bp insertions, 100 bp inversion, 100 bp translocation, and 100 kbp deletion were introduced into each chromosome at position 1,000,001, 2,000,001, 3,000,001, 4,000,001, 5,000,001, 6,000,001, 7,000,001, 8,000,001, and 9,000,001, respectively. Short-reads with 100 bp length were sliced at 2 bp intervals from the artificial genome sequence. Paired complementary reads were sliced at a position 231 bp forward. Single nucleotide mutations were introduced at 0.1% frequency as artificial read errors of the next-generation sequencer, and then formatted to fastq format. For fastq file of control reads, reads were sliced from the original reference sequence by the same method.

### Mutation detection by pipeline of bwa, Samtools and GATK

The artificial rice genome was analyzed by our methods, Samtools and GATK. For Samtools and GATK, short-reads were mapped by bwa 0.7.16a-r1181. The version of Samtools and GATK was 1.6 and 4.0.2.1, respectively. Vcf files were obtained by mpileup command for Samtools and HaplotypeCaller command for GATK.

### Drawing graphical genotype map

Maps for positions of SNPs from the bidirectional alignment and bwa + GATK were drawn by a perl script with GD module.

### Detection of deletions by PCR

Human genomic DNA (10 ng) from Coriell Institute were amplified by EmeraldAmp (TaKaRa Bio Inc.) and 0.2 μM primers with 35 thermal cycles at 94 °C for 30 s, 60 °C for 30 s, and 72 °C for 30 s. Reaction mixtures were electrophoresed with 3% agarose gel in Tris-Borate-EDTA buffer. Gel was stained with 0.5 μg/ml ethidium bromide and then products were visualized with 254 nm ultraviolet light. Primer sequences for 144 bp deletion from 49,334,346 to 49,334,487 on chromosome 2 in NA12878 (ERR194147) were CAGGTCTCCAACAGTTTCCC (P1F) and TGGTGGGAATAGAGTGGAGC (P1R). Primer sequences for 35,579 bp deletion from 52,522,549 to 52,558,132 on chromosome 2 in NA12878 are CTGCATGCATTTACACTGGG (P2F) and CAGCTTGCAGAACCCTAAGC (P2R). For detection of non-deleted allele, primers with the nucleotide sequences CAGTAAGCTCCCCAAAGCTG (P3F) and CAAGCATTCCCTGGAAGGTA (P3R) were used. For detection of the deletion from 9,974,386 to 9,975,667 on chromosome 9 in ‘Koshihikari’, primer pair of TATCCGAGCAATGCACCAAC (P4F) and TGTTTCGCTTCATTGCTGGG (P4R) are used.

### Confirmation of SNP by dye-terminator sequence

A SNP which did not appear in the high-confidence variant call for NA12878 by Illumina was selected from the segregated SNPs among our CEPH pedigree 1463 data. Primer sequences to amplify A to C mutation at position 115,921,403 in chromosome 1 were ATCTGGGTGGTGGTTGTACC and CGTGTGATAAGCATGTGGATT. PCR conditions were the same as for the detection of deletions. Products amplified with genome DNA of NA12877 and NA12878 were separated on 2% agarose gel, stained with ethidium bromide, and visualized with 312 nm ultraviolet light. Gel pieces containing fragments were then sliced. Fragments on agarose gel were purified with Wizard SV Gel and PCR Clean-Up system (Promega A9282) and then sequenced using BigDye Terminator v3.1 Cycle Sequencing Kit (Applied Biosystems 4,337,455) and DNA Analyzers (Applied Biosystems 3730xl).

## Additional files

Additional file 1: **Figure S1.** An example of bidirectional alignment. SNP was detected by the single-directional alignment. Insertion, deletion, inversion, and translocation were detected by the bidirectional alignment. The polymorphic edge, i.e.*,* the mismatch detected first, is indicated with a vertical bar and location on the chromosome. Summarized data following # are the order of chromosome number and position of the edge detected by matching from the 5′-end, chromosome number and position from the 3′-end, direction, type of polymorphism, and size of insertion or deletion. The next line of the summarized data is the sequence of *k*-mers for detection of map location of 5′- and 3′- ends following the size of the margin described in ‘Mapping by join command’ section in Methods. (PPTX 30 kb)

Additional file 2: **Table S1.** Detection performance of SNP by artificially-modified rice genome. (XLSX 13 kb)

Additional file 3: **Table S2.** Detection performance of structural variation by artificially-modified rice genome. (XLSX 13 kb)

Additional file 4: **Table S3.** Detection of SNPs introduced during cancer cell development by *k*-mer method. (XLSX 169 kb)

Additional file 5: Scripts for benchmark. (TXT 2 kb)

## Data Availability

The PED software is available at: https://github.com/akiomiyao/ped. Results are reproducible using PED software and public sequence data which is described in methods section. Rice genomic DNA are available from National Agriculture and Food Research Organization.

## Abbreviations

NGS: Next generation sequencing
PED: Polymorphic edge detection
SNP: Single nucleotide polymorphism
SV: Structural variation
