# Supplementary material for: Polymorphic edge detection (PED): two efficient methods of polymorphism detection from next-generation sequencing data
Source: BMC Bioinformatics. 2019 Jun 28;20:362. doi: 10.1186/s12859-019-2955-6 (PMC6599308; doi:10.1186/s12859-019-2955-6)
Supplement: Supplementary file 1 — Figure S1. An example of bidirectional alignment. SNP was detected by the single-directional alignment. Insertion, deletion, inversion, and translocation were detected by the bidirectional alignment. The polymorphic edge, i.e., the mismatch detected first, is indicated with a vertical bar and location on the chromosome. Summarized data following # are the order of chromosome number and position of the edge detected by matching from the 5′-end, chromosome number and position from the 3′-end, direction, type of polymorphism, and size of insertion or deletion. The next line of the summarized data is the sequence of k-mers for detection of map location of 5′- and 3′- ends following the size of the margin described in ‘Mapping by join command’ section in Methods. (PPTX 30 kb) [file 12859_2019_2955_MOESM1_ESM.pptx]

## Slide 1
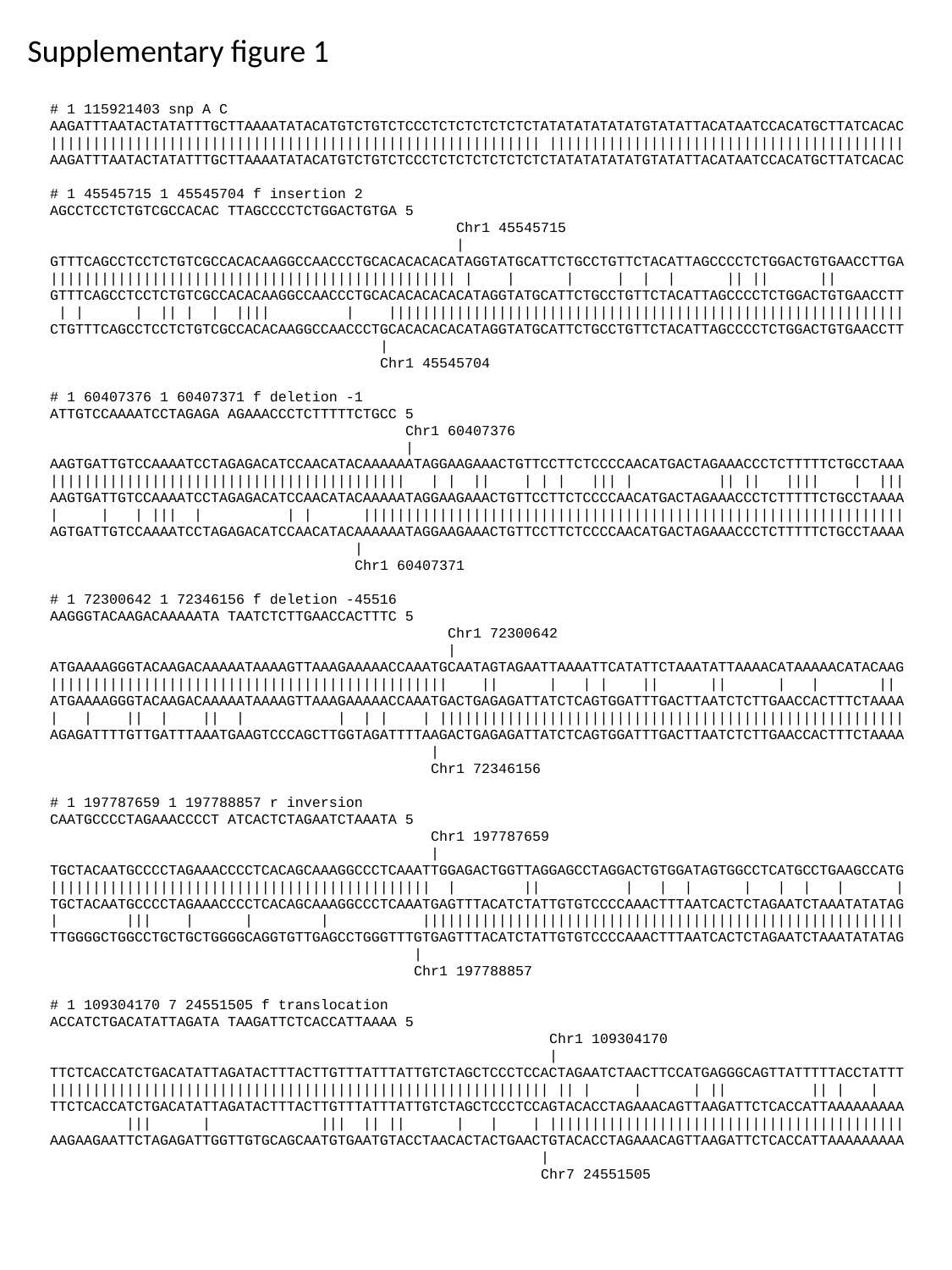

Supplementary figure 1
# 1 115921403 snp A C
AAGATTTAATACTATATTTGCTTAAAATATACATGTCTGTCTCCCTCTCTCTCTCTCTATATATATATATGTATATTACATAATCCACATGCTTATCACAC
|||||||||||||||||||||||||||||||||||||||||||||||||||||||||| ||||||||||||||||||||||||||||||||||||||||||
AAGATTTAATACTATATTTGCTTAAAATATACATGTCTGTCTCCCTCTCTCTCTCTCTCTATATATATATGTATATTACATAATCCACATGCTTATCACAC
# 1 45545715 1 45545704 f insertion 2
AGCCTCCTCTGTCGCCACAC TTAGCCCCTCTGGACTGTGA 5
 Chr1 45545715
 |
GTTTCAGCCTCCTCTGTCGCCACACAAGGCCAACCCTGCACACACACATAGGTATGCATTCTGCCTGTTCTACATTAGCCCCTCTGGACTGTGAACCTTGA
|||||||||||||||||||||||||||||||||||||||||||||||| | | | | | | || || ||
GTTTCAGCCTCCTCTGTCGCCACACAAGGCCAACCCTGCACACACACACATAGGTATGCATTCTGCCTGTTCTACATTAGCCCCTCTGGACTGTGAACCTT
 | | | || | | |||| | |||||||||||||||||||||||||||||||||||||||||||||||||||||||||||||
CTGTTTCAGCCTCCTCTGTCGCCACACAAGGCCAACCCTGCACACACACATAGGTATGCATTCTGCCTGTTCTACATTAGCCCCTCTGGACTGTGAACCTT
 |
 Chr1 45545704
# 1 60407376 1 60407371 f deletion -1
ATTGTCCAAAATCCTAGAGA AGAAACCCTCTTTTTCTGCC 5
 Chr1 60407376
 |
AAGTGATTGTCCAAAATCCTAGAGACATCCAACATACAAAAAATAGGAAGAAACTGTTCCTTCTCCCCAACATGACTAGAAACCCTCTTTTTCTGCCTAAA
|||||||||||||||||||||||||||||||||||||||||| | | || | | | ||| | || || |||| | |||
AAGTGATTGTCCAAAATCCTAGAGACATCCAACATACAAAAATAGGAAGAAACTGTTCCTTCTCCCCAACATGACTAGAAACCCTCTTTTTCTGCCTAAAA
| | | ||| | | | ||||||||||||||||||||||||||||||||||||||||||||||||||||||||||||||||
AGTGATTGTCCAAAATCCTAGAGACATCCAACATACAAAAAATAGGAAGAAACTGTTCCTTCTCCCCAACATGACTAGAAACCCTCTTTTTCTGCCTAAAA
 |
 Chr1 60407371
# 1 72300642 1 72346156 f deletion -45516
AAGGGTACAAGACAAAAATA TAATCTCTTGAACCACTTTC 5
 Chr1 72300642
 |
ATGAAAAGGGTACAAGACAAAAATAAAAGTTAAAGAAAAACCAAATGCAATAGTAGAATTAAAATTCATATTCTAAATATTAAAACATAAAAACATACAAG
||||||||||||||||||||||||||||||||||||||||||||||| || | | | || || | | ||
ATGAAAAGGGTACAAGACAAAAATAAAAGTTAAAGAAAAACCAAATGACTGAGAGATTATCTCAGTGGATTTGACTTAATCTCTTGAACCACTTTCTAAAA
| | || | || | | | | | |||||||||||||||||||||||||||||||||||||||||||||||||||||||
AGAGATTTTGTTGATTTAAATGAAGTCCCAGCTTGGTAGATTTTAAGACTGAGAGATTATCTCAGTGGATTTGACTTAATCTCTTGAACCACTTTCTAAAA
 |
 Chr1 72346156
# 1 197787659 1 197788857 r inversion
CAATGCCCCTAGAAACCCCT ATCACTCTAGAATCTAAATA 5
 Chr1 197787659
 |
TGCTACAATGCCCCTAGAAACCCCTCACAGCAAAGGCCCTCAAATTGGAGACTGGTTAGGAGCCTAGGACTGTGGATAGTGGCCTCATGCCTGAAGCCATG
||||||||||||||||||||||||||||||||||||||||||||| | || | | | | | | | |
TGCTACAATGCCCCTAGAAACCCCTCACAGCAAAGGCCCTCAAATGAGTTTACATCTATTGTGTCCCCAAACTTTAATCACTCTAGAATCTAAATATATAG
| ||| | | | |||||||||||||||||||||||||||||||||||||||||||||||||||||||||
TTGGGGCTGGCCTGCTGCTGGGGCAGGTGTTGAGCCTGGGTTTGTGAGTTTACATCTATTGTGTCCCCAAACTTTAATCACTCTAGAATCTAAATATATAG
 |
 Chr1 197788857
# 1 109304170 7 24551505 f translocation
ACCATCTGACATATTAGATA TAAGATTCTCACCATTAAAA 5
 Chr1 109304170
 |
TTCTCACCATCTGACATATTAGATACTTTACTTGTTTATTTATTGTCTAGCTCCCTCCACTAGAATCTAACTTCCATGAGGGCAGTTATTTTTACCTATTT
||||||||||||||||||||||||||||||||||||||||||||||||||||||||||| || | | | || || | |
TTCTCACCATCTGACATATTAGATACTTTACTTGTTTATTTATTGTCTAGCTCCCTCCAGTACACCTAGAAACAGTTAAGATTCTCACCATTAAAAAAAAA
 ||| | ||| || || | | | ||||||||||||||||||||||||||||||||||||||||||
AAGAAGAATTCTAGAGATTGGTTGTGCAGCAATGTGAATGTACCTAACACTACTGAACTGTACACCTAGAAACAGTTAAGATTCTCACCATTAAAAAAAAA
 |
 Chr7 24551505
